# Evolution of opercle bone shape along a macrohabitat gradient: species identification using mtDNA and geometric morphometric analyses in neotropical sea catfishes (Ariidae)

**DOI:** 10.1002/ece3.2334

**Published:** 2016-07-22

**Authors:** Madlen Stange, Gabriel Aguirre‐Fernández, Richard G. Cooke, Tito Barros, Walter Salzburger, Marcelo R. Sánchez‐Villagra

**Affiliations:** ^1^ Palaeontological Institute and MuseumUniversity of ZurichKarl‐Schmid‐Strasse 48006ZurichSwitzerland; ^2^ Zoological InstituteUniversity of BaselVesalgasse 14051BaselSwitzerland; ^3^ Smithsonian Tropical Research InstituteMRC 0580‐08Apartado0843‐03092PanamaRepublic of Panama; ^4^ Museo de BiologíaFacultad Experimental de CienciasLa Universidad del ZuliaApartado Postal 526Maracaibo4011Estado ZuliaVenezuela

**Keywords:** Geometric morphometrics, macrohabitat transition, mitochondrial DNA, Siluriformes, systematics, taxonomy

## Abstract

Transitions between the marine and freshwater macrohabitat have occurred repeatedly in the evolution of teleost fishes. For example, ariid catfishes have moved from freshwater to marine environments, and vice versa. Opercles, a skeletal feature that has been shown to change during such transitions, were subjected to 2D geometric morphometric analyses in order to investigate evolutionary shape changes during habitat transition in ariid catfishes and to test the influence of habitat on shape changes. A mtDNA marker, which proved useful in previous studies, was used to verify species identities. It greatly improved the assignment of specimens to a species, which are difficult to assign by morphology alone. The application of a mtDNA marker confirmed the occurrence of *Notarius biffi* in Central America, South of El Salvador. Molecular identification together with principal component analysis (PCA) and further morphological inspection of neurocrania indicated the existence of a cryptic species within *Bagre pinnimaculatus*. Principal component (PC) scores of individual specimens clustered in morphospace by genus rather than by habitat. Strong phylogenetic structure was detected using a permutation test of PC scores of species means on a phylogenetic tree. Calculation of Pagel's *λ* suggested that opercle shape evolved according to a Brownian model of evolution. Yet canonical variate analysis (CVA) conducted on the habitat groups showed significant differences in opercle shapes among freshwater and marine species. Overall, opercle shape in tropical American Ariidae appears to be phylogenetically constrained. This verifies the application of opercle shape as a taxonomic tool for species identification in fossil ariid catfishes. At the same time, adaptation to freshwater habitats shows characteristic opercle shape trajectories in ariid catfishes, which might be used to detect habitat preferences in fossils.

## Introduction

The colonization of freshwater habitats by marine ancestors (and vice versa) is a common scenario in the evolution of fishes and has occurred multiple times in various teleost families, including Gasterosteidae (sticklebacks) (Bell and Foster 1994), Belonidae (needlefish) (Lovejoy et al. 2006), Atherinopsidae (neotropical silversides) (Bloom et al. 2013), and Terapontidae (grunters) (Davis et al. 2012), as well as in elasmobranchs, such as Myliobatiformes (stingrays) (Lovejoy et al. 2006) and Carcharhinidae (requiem sharks) (de Carvalho, M.R., McEachran, J.D., 2003). Such transitions from marine to freshwater macrohabitats implicate varied adaptations to different environmental conditions in, for example, salinity, pH, flow conditions, oxygen content, and feeding ecology. Here, we investigated the opercle, a skeletal feature of teleosts that is not actively involved in the adaptation to any of these changes but may be indirectly affected, and hence should reflect adaptations to a variety of environmental changes. The opercle is positioned laterally and centrally on the head, and changes dimensions according to skull shape. Its primary function is the protection of the gills, which make it useful for study as it is present in most teleost fishes, it can be easily examined from the outside, and it is often well preserved in the fossil record. Therefore, the study of the opercle allows comparisons both across teleosts in general and in deep time (see, e.g., Wilson et al. 2015).

Opercle shape has been studied in different contexts in various fish taxa, including extant Alaskan *Gasterosteus aculeatus* (stickleback) populations, Antarctic notothenioids, Lake Tanganyika cichlids, and extinct *Saurichthys* species (Kimmel et al. 2008; Wilson et al. 2013a,b, 2015); the development of the opercle has further been studied in *Danio rerio* (zebrafish) (Kimmel et al. 2010) and in *Saurichthys* (Scheyer et al. 2014). Overall, these studies have revealed a strong link between the shape of the opercle and the habitat and/or feeding type of the respective species. For example, Alaskan sticklebacks that invaded lake environments diverged from their anadromous ancestors in opercle shape (Kimmel et al. 2005, 2012a; Arif et al. 2009) with the opercle of lake populations being stretched along the anterior–posterior axis, while compressed along the dorsal–ventral axis (Kimmel et al. 2008). The characteristic shape of the opercle in freshwater specimens is likely due to a developmental decoupling of shape and size, as freshwater populations retain the juvenile ancestral opercle shape despite achieving full body size (Kimmel et al. 2012b). Indeed, opercle size and shape have previously been demonstrated to arise from two independent developmental modules in *D. rerio* (Kimmel et al. 2010). Divergence in opercle shape has further been shown between inhabitants of shallow and deep Alaskan lakes (Arif et al. 2009). Among extinct marine *Saurichthys* species, a dorsoventral compression could be observed (Wilson et al. 2013b). Similar to *G*. *aculeatus*, but slightly offset, an anterior–posterior stretching and dorsal–ventral compression was observed in Antarctic notothenioid fishes (Wilson et al. 2013a). The notothenioids, which diverged between 17.1 and 10 mya (Colombo et al. 2015), represent a relatively old adaptive radiation, especially when compared to sticklebacks. Another pattern observed in notothenioids is that opercle shape is strongly influenced by phylogeny, although within subfamilies, opercle shapes reflect adaptations along the benthic–pelagic axis (Wilson et al. 2013a). In the cichlid adaptive radiation in Lake Tanganyika, a significant correlation between opercle shape and gut length was uncovered (Wilson et al. 2015), suggesting that opercle shape reflects adaptations in feeding ecology. In summary, two major factors have been identified to contribute to opercle shape: habitat and phylogeny (Wilson et al. 2015).

Here, we investigated opercle shape in neotropical sea catfishes of the “family” Ariidae and the influence of habitat and phylogeny upon it. Ariid catfishes belong to an exceptional fish radiation (Alfaro et al. 2009) that diversified along a marine–freshwater habitat trajectory. These fish may therefore show evidence of opercle shape evolution along this macrohabitat axis, comprising adaptation events in recent as well as in geological times. Siluriformes (catfishes) diverged sometime between 180 mya (Nakatani et al. 2011 [molecular data]) and before the Late Cretaceous (more than 100 mya) (Diogo 2004 [fossil evidence]). Today the Siluriformes consist of more than 3000 species [www.fishbase.org, version 01/2016]) in 33 “families”, with most of the catfish species being primary freshwater inhabitants (Teugels 1996). Some “families” contain species with a preference for brackish habitats, such as the Loricariidae and Pimelodidae (Betancur‐R 2009, 2010), but only two of the 33 “families” – including the widely distributed Ariidae – can be characterized as primarily marine. Nonetheless, some members of the Ariidae have secondarily colonized freshwater habitats, so that Ariidae inhabit the coastal waters and near‐coastal rivers and lakes of most tropical and subtropical regions worldwide (Sullivan et al. 2006). The evolutionary history of Ariidae has been relatively well traced in tropical America, as some structures such as the lapillus (largest ear stones in Ariidae), spines, and partial skulls preserve rather well in the fossil record. All these features contain taxonomic information (Aguilera et al. 2013), with the lapillus in particular allowing identification to the genus and in some cases species level (Acero and Betancur‐R 2007 and references therein; Chen et al. 2011).

A habitat change from marine to freshwater, as recorded in the Ariidae, was a fundamental part of the history of other South American biota, including dolphins, stingrays, and needlefish (Lovejoy et al. 2006). During a major event in the Miocene (23–5 mya), sea level differences and tectonic activities shaped and reshaped the aquatic landscape on this continent, forming the Amazon River (Hoorn and Hoorn 2006; Lovejoy et al. 2006). Some tropical American ariid species are known to be present in their respective habitats since the lower Miocene (23 mya) (Aguilera and de Aguilera 2004). Other ariid species might have taken the opportunity to occupy new habitats during these major geological events. Indeed, it is likely that the Ariidae still have the propensity to occupy freshwater habitats as it has been observed in *Cathorops tuyra* in Panama (D. Sharpe, pers. comm., December, 2015). During our sampling, we found species in habitats and localities in which they had not been reported to occur. For that reason, we summarized the species we found in each habitat and substantiated the correctness of species identity by the usage of a mtDNA marker for comparison with a reference dataset.

We investigated habitat transition in a group of catfishes by providing information on distribution and taxonomy as examined by molecular markers in a phylogenetic context and based on shape analysis of the opercle bone as morphological marker. Unlike Antarctic notothenioids, Ariidae show no subgroup divergence within genera. Nonetheless, opercle shapes between marine and freshwater groups are significantly different. The same pattern of dorsal–ventral compression and anterior–posterior stretching along the two major axes of variance was detected as in the *Gasterosteus aculeatus* (stickleback), Antarctic notothenioids, and Lake Tanganyika cichlids, suggesting this to be a general trend in teleosts.

## Methods

### Sampling and species Identification using the mtDNA marker *ATPase 8/6*

**Figure 1:**
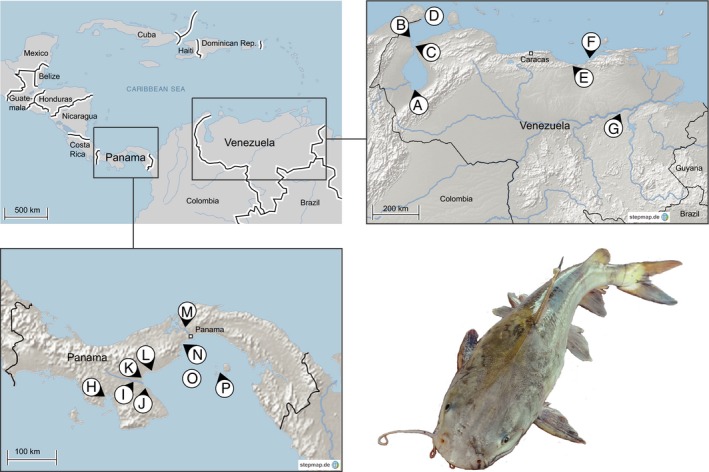
Map of sampling locations. The fish picture depicts a representative of *Cathorops*. A: Puerto Concha, Maracaibo Lake, Zulia state, VE. B: Isla de Toas and Isla de San Carlos, Maracaibo Lake, Zulia state, VE. C: Guarico, Maracaibo Lake, Zulia state, VE. D: Gulf of Venezuela, Falcón state, VE. E: Clarines, Anzoátegui state, VE. F: Puerto la Cruz, Anzoátegui state, VE. G: Ciudad Bolivar, Bolivar state, VE. H: Río San Pedro, Montijo Bay, PA. I: Rio Santa Maria, PA. J: Rio Parita, PA. K: Rio Estero Salado, PA. L: Rio Hato, PA. M: Rio Chagres, PA. N: Puerto Caimito, PA. O: Gulf of Panama, PA. P: Pearl Islands, Casaya Island, PA. For additional information, see Tables S1 and S3.

Twenty‐two ariid catfish species belonging to the genera *Bagre*,* Sciades*,* Cathorops*,* Notarius*, and *Ariopsis* were sampled in marine, brackish, and freshwater habitats of Venezuela (VE) and Panama (PA) (Fig. 1). GPS coordinates, sampled species in each location, and habitat definition can be found in Table S1. In Venezuela, specimens were bought fresh from local fish markets, or from hired fishers, while in Panama specimens were caught directly by the authors with the support of local fishers. Taxonomic identifications of all specimens were conducted in the field on the basis of characteristics of morphological traits, for example, neurocranium shape (visible through the skin), color, dentary morphology and, after maceration, neurocrania again (Fischer et al. 1995; Acero 2002; Betancur‐R and Acero 2005; Marceniuk 2007; Lasso and Sánchez‐Duarte 2011; Marceniuk et al. 2012). The sampled material included caudal fin tissue preserved in 96% ethanol for DNA isolation, and macerated and bleached opercle bones. For verification of the taxonomic affiliation, sequencing of a mtDNA marker was conducted.

In total, 263 ariid catfishes were sampled, and isolated DNA (for unique sample identifiers with species, locality information and NCBI accession numbers, see Table S3) was subjected to standard Sanger sequencing targeting mitochondrial *ATPase 8/6*. DNA was isolated using standard salt precipitation. For details on amplification and sequencing, see Appendix S1. Sequence traces (available on NCBI, accession numbers KX500399‐KX500661) were inspected for quality control, removing poor‐quality bases and/or ambiguous base calls using CodonCode Aligner v.3.7.1.1 (CodonCode, Dedham, MA). These were aligned using MAFFT v.7 (Katoh and Standley 2013) together with a reference dataset (Betancur‐R 2009) containing 281 *ATPase 8/6* sequences from 129 ariid species distributed worldwide. This reference dataset was kindly provided by R. Betancur‐R. Maximum‐likelihood trees were constructed using RAxML 7.0.3 (Stamatakis 2006) under the GTR+I+G model. The best tree from 1000 bootstrap replicates was used to infer molecular species identity by clustering of our sampled specimens to the reference sequences of species.

### 2D geometric morphometric (GM) data collection and analyses

**Figure 2:**
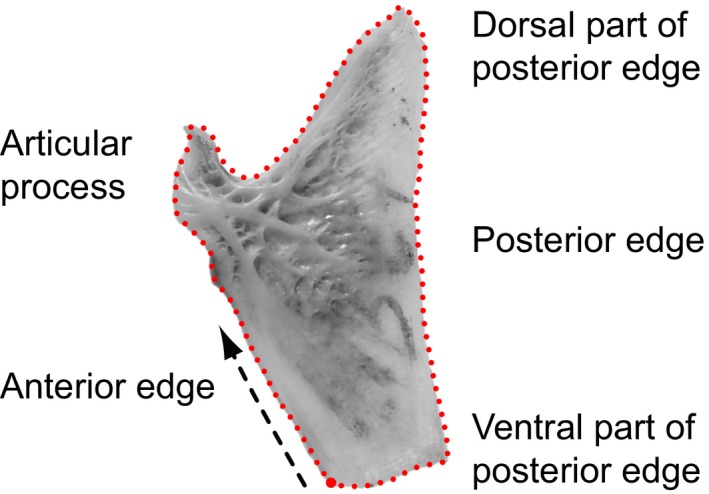
One hundred equidistant landmarks on a left ariid opercle (*Notarius quadriscutis*). The starting and end point of landmark capturing is indicated by a bold red dot, representing a Type 2 landmark, the local maximum of the curvature on the ventral part of the anterior edge. Ninety‐nine sliding semilandmarks were equally spaced capturing the outline of the opercle bone. Direction of landmark recording along the outline is indicated by an arrow.

Opercle bones of 263 specimens (deposited at the Palaeontological Institute and Museum of the University of Zurich, PIMUZ; for unique identifiers, see Table S3) were subjected to GM analysis. Left and right opercles (if present) of each specimen were photographed from lateral view using a digital camera mounted on a table stand. Raw images of the right opercle were reorientated and reflected in Adobe Photoshop CS6 to match left opercle orientation. This is necessary for merging the shape data of left and right opercle in downstream analyses. All raw images were further processed using the TpsUtil v.1.60 software (http://life.bio.sunysb.edu/morph/soft-utility.html). In order to analyze the shape of the opercle bone, a sliding semilandmark approach was applied. The first landmark was defined as the most ventral point of the anterior edge and represents the only true or fixed landmark (Type 2 landmark) in this study. Ninety‐nine equidistant points (Fig. 2) were then placed, capturing the outline of each opercle using TpsDig v2.10 (Rohlf 2013). This approach was chosen above a true landmark approach, as the opercle shapes among genera varied greatly and prohibited the identification of further homologous landmarks. During Procrustes superimposition, performed in the R package geomorph v.3.0.0 (Adams and Otarola‐Castillo 2013), differences in scale, orientation, and size were removed, and each semilandmark was moved along its tangent vector (between its left and right neighboring semilandmark) until its position minimized the shape difference between specimens based on bending energy (Bookstein 1997; Bookstein et al. 1999; Gunz et al. 2005; Rohlf 2010). The slid and superimposed landmark coordinates were imported to MorphoJ 1.06d (Klingenberg 2011), where all subsequent morphometric analyses were conducted.

The shape differences between left and right opercle bones within specimens were assessed using Procrustes ANOVA on geometric shape data (Klingenberg et al. 2002). Interspecimen shape differences were much larger than intraspecimen shape differences (within‐specimen Procrustes sum of squares = 0.0021 [*F* = 1.9, *P* = <0.0001]; between‐specimen Procrustes sum of squares = 2.88 [*F* = 28.24, *P* = <0.0001]). Therefore, we averaged shape data (Procrustes coordinates and centroid size) of left and right opercle by specimen for downstream analyses if both the left and right opercles were available (*n *= 250), or included shape data for single opercles if only one opercle was available (*n* = 13).

Allometric effects within species, caused by specimens of different ontogenetic stages, were removed by regressing pooled within‐species Procrustes coordinates (shape) onto centroid size (Drake and Klingenberg 2008). The resulting regression residuals were then used to calculate a covariance matrix for the subsequent multivariate analyses.

### Patterns of opercle shape variation among ariid species

Principal component analyses (PCAs) were used to identify axes of maximal shape variance among all ariid specimens in order to discover patterns of variation, to explore groupings among them, and to assess phylogenetic structure in the groupings.

**Figure 3:**
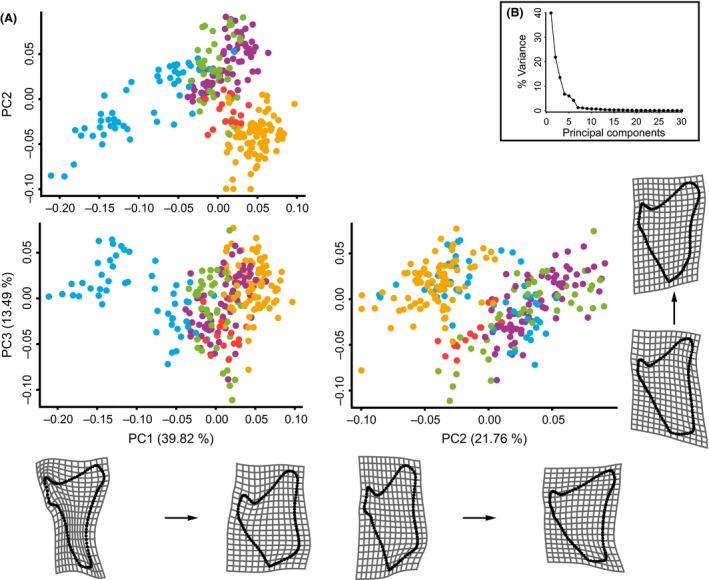
Patterns of ariid opercle shape variation along major axes of variance. (A) Scatterplots of the first three principal components (PCs) of ariid opercle shapes (*N* = 263) from 21 species belonging to the genera *Bagre* (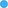), *Sciades* (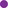), *Cathorops* (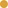), *Notarius* (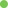), and *Ariopsis* (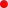). A detailed scatterplot of species can be found in Figure S1. The opercle shape changes are displayed below or next to the respective axes of variance using thin plate spline visualization grids using the starting shape at scale factor 0 as reference shape. The respective shape changes are scaled by −0.2 and +0.1 (magnitude of shape change in Procrustes distance) for PC1, −0.1 and +0.1 for PC2, and −0.1 and +0.07 for PC3. (B) Scree plot of the first 30 PCs and their amount of variance. The bent after the third PC indicates a distinct drop in the impact of following PCs on shape variance. Therefore, only the first three PCs are presented in the scatterplot. The first three PCs together account for 75.07% of the observed variation.

**Figure 4:**
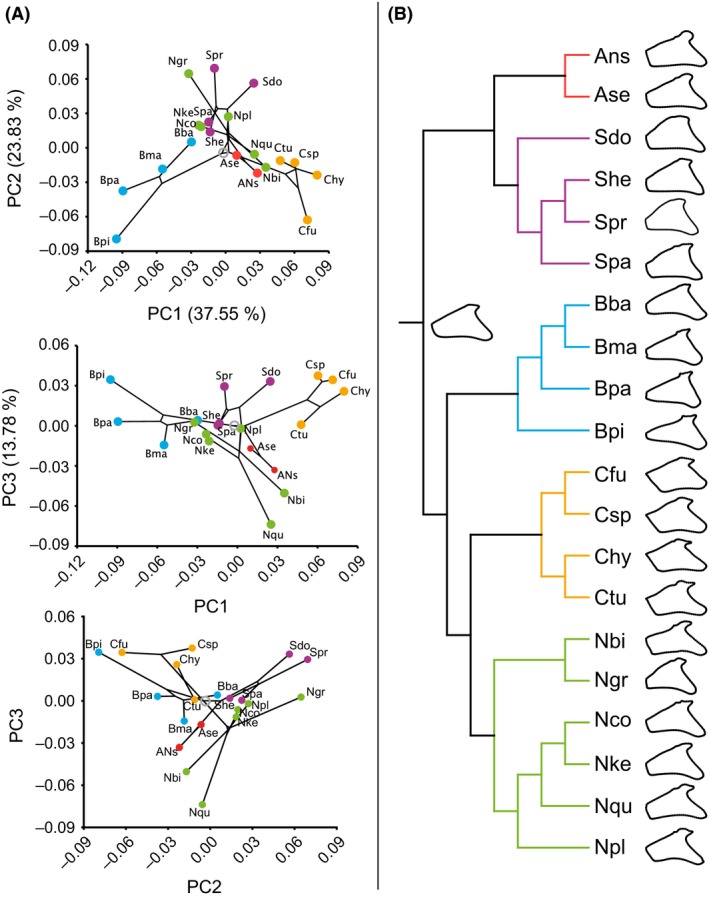
Evolutionary opercle shape change within Ariidae. (A) Phylomorphospace plot of ariid opercle shape changes. A time‐calibrated tree constructed from mitochondrial and nuclear markers published by Betancur‐R et al. (2012) has been projected on PC scores derived from species means. The first three principal components account for 75.16% of the total variation. PC scores of 20 species are displayed and highlighted by genus (*Bagre* (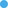), *Sciades* (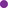), *Cathorops* (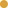), *Notarius* (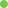), and *Ariopsis* (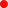), root (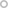)). Ans: *A*. sp. nov. Ase: *A. seemanni*; Sdo: *S. dowii*; She: *S. herzbergii*; Spr: *S. proops*; Spa: *S. parkeri*; Bba: *B. bagre*; Bma: *B. marinus*; Bpa: *B. panamensis*; Bpi: *B. pinnimaculatus*; Cfu: *C. fuerthii*; Csp: *Cathorops* sp. (includes *C. wayuu* and *C. nuchalis*, as both species do not differ in the 11 genes analyzed by Betancur‐R et al.); Chy: *C. hypophthalmus*; Ctu: *C. tuyra*; Nbi: *N. biffi*; Ngr: *N. grandicassis*; Nco: *N. cookei*; Nke: *N. kessleri*; Nqu: *N. quadriscutis*; Npl: *N. planiceps*. PC scores for *C*. sp. indet. are not displayed, as they are not included in the phylogeny of Betancur‐R et al. (2004). The permutation of shapes along the phylogeny resulted in a *P*‐value of 0.001 rejecting the null hypothesis of absence of phylogenetic signal. Pagel's *λ* of 1 is not significantly different from 1 (*P* = 1) implying opercle shape evolution happened according to the Brownian model of evolution. (B) Evolutionary opercle shape change along the time‐calibrated tree (Betancur‐R et al. 2012) that has been projected on Procrustes coordinates derived from species means. Mean shapes per species are displayed at the node tips, and the ancestral opercle state for the shapes studied here is displayed at the first internal split at the root.

In a first PCA (Fig. 3), principal component (PC) scores for all 263 samples were calculated and plotted to investigate the clustering behavior of our samples in morphospace. The number of individuals per species and locality is listed in Table S2. Subsequently, we tested for phylogenetic structure in the shape data by calculating centroid sizes and Procrustes coordinates, representing species means, in order to match shape and size values with the terminal taxa in the phylogenetic tree. A new covariance matrix was calculated, a second PCA was performed, and a time‐calibrated tree constructed from mitochondrial and nuclear markers, published by Betancur‐R et al. (2012), was mapped onto the PC scores (Fig. 4A). This time‐calibrated tree (Betancur‐R et al. 2012) represents the currently most resolved phylogenetic tree of the Ariidae. Although the tree contains a large number of species, not all species analyzed here are contained, forcing us to average shape data of *C. nuchalis* and *C. wayuu* to *Cathorops* sp. This restriction also hindered our analysis of within‐species shape differentiation (among habitats) as data for freshwater and marine populations had to be merged for phylogenetically corrected shape analyses. A total of 1000 permutations simulating the null hypothesis of total absence of phylogenetic structure by randomly permuting the PC scores among the species and mapping onto the phylogeny were applied (Klingenberg and Gidaszewski 2010). The resulting *P*‐value represents the proportion of permutations that resulted in equal or shorter tree lengths than the input tree. To additionally test for phylogenetic dependence on the species trait values, in this case PC scores per species, a phylogenetic least squares (PGLS) analysis (Martins and Hansen 1997) was performed using a maximum‐likelihood estimate of Pagel's *λ*, implemented in the R package “Caper” (Orme et al. 2013). This test assumes a Brownian model of evolution, where variation between tips along all branches of the tree accumulates at a rate proportional to the length of the branches, meaning that the more closely that taxa are related, the more similar they are in trait values, and vice versa (*λ* = 1). A trait value evolution that disagrees with the Brownian model would result in *λ* = 0. The evolutionary opercle shape change and ancestral shape were reconstructed by projecting the phylogeny on Procrustes coordinates of species means (Fig. 4B).

### Opercle shape variation according to habitat

Canonical variate analysis (CVA) was used to visualize opercle shape changes that discriminate among the different habitats of our sampled specimens. CVA computes axes of variance in a way that minimized within‐group differences and maximized between‐group differences. Samples were assigned a priori to the following groups: freshwater (*n* = 38, 5 species), brackish (*n* = 94, 14 species), or marine (*n* = 131, 8 species). For numbers of individuals used per species and habitat, see Table S2. As the precise habitat range of most species is unknown, the classification as freshwater‐, brackish‐, or marine‐occurring species follows the habitat where fishers or the authors captured the specimens. Therefore, individuals of the same species were in some cases assigned to different habitats. As in the PCA, the residuals of the within‐group regression of Procrustes coordinates onto centroid size per specimen were used (in order to correct data for allometry). The significance of differences among group means (habitats) was tested in a permutation test with 1000 random permutations, and distances are presented in Procrustes and Mahalanobis measures.

## Results

### Species identification using the mtDNA marker *ATPase 8/6*

The morphological and molecular assignment of specimens was not concurrent in all cases. By aligning gained *ATPase 8/6* sequences against a reference dataset, 19 ariid species instead of 21, from five genera, were confirmed. Four individuals had identical sequences but could not be assigned to any specific species, using either morphological or molecular data. These sequences showed close affinity to *Cathorops*. The individuals were therefore labeled as *C*. sp. indet. Some species that originated from different localities exhibited distinct *ATPase 8/6* sequences; others had identical sequences despite different sampling localities, and still others were found in unreported habitats. The results are summarized in Table 1 (see also Appendix S2).

**Table 1:** Species identification using a mtDNA marker *ATPase 8/6*

Genus	Species	Localities^a^	Notes
*Sciades*	*proops* (Valenciennes, 1840)	D^m^ (17), E^m^ (1), F^m^ (1)	Two sequence clusters corresponding to sampling locations, E and F, differ from D
	*herzbergii* (Bloch, 1784)	D^m^ (31), E^m^ (15)	Two sequence clusters corresponding to sampling locations
	*dowii* (Gill 1863)	Mouth of L^b^ (1), N^m^ (2), I^f^ (2)	No sequence difference according to sampling location
	*parkeri* (Traill 1832)	G^f^ (2)	This species is described as occurring in lower parts of rivers from the Gulf of Paria, VE, to Brazil (Betancur‐R et al. 2008). In this study, the species was found 320 km inland
*Ariopsis*	*seemanni* (Günther 1864)	K^b^ (5)	
	sp. nov. (*A. jimenezi*, A. Marceniuk et al., in prep.)	P^m^ (9)	
*Notarius*	*cookei* (Acero and Betancur‐R 2002)	I^f^ (7)	Described as brackish water species (Betancur‐R et al. 2007). In this study, it was sampled in freshwater, 10 km upstream from the mouth of the Santa Marias river. Vega (pers. comm., Dec. 2015) captured it in the same river at locations between 69 and 76 lm from the mouth in completely fresh water
	*kessleri* (Steindachner 1876)	H^b^ (8), K^b^ (2)	No sequence difference according to sampling location
	*biffi* (Betancur‐R and Acero 2004)	H^b^ (1)	First reported as restricted from El Salvador to Costa Rica (Betancur‐R and Acero 2004), but see Robertson, 2015 who presented records from western and central Panama to Parita Bay. We sampled it in the Gulf of Montijo, Panama
	*quadriscutis* (Valenciennes, 1840)	E^b^ (10)	
	*grandicassis* (Valenciennes, 1840)	D^m^ (11)	
	*planiceps* (Steindachner, 1877)	K^b^ (1)	
*Bagre*	*bagre* (Linnaeus, 1766)	D^m^ (3), B^b^ (2)	No sequence difference according to sampling location.
	aff. *marinus*	D^m^ (16), F^m^ (3)	No sequence difference according to sampling location
	*panamensis* (Gill 1863)	K^b^ (3)	
	*pinnimaculatus* (Steindachner 1876)	K^b^ (1), O^m^ (4, atypical: 20)	Several specimens (atypical) from the Gulf of Panama (O) diverged from the morphology of *B. pinnimaculatus* by lacking the typical hyperossification of the frontals, preopercle, and interopercle. They did not exhibit the phenotype of *B. panamensis*, either, the only other *Bagre* species occurring in the eastern Pacific. Morphologically they resembled *B. bagre* from the Atlantic. In the ML tree, those specimens formed a sister clade to *B. pinnimaculatus*. However, due to their molecular relatedness they were considered being *B. pinnimaculatus* in the subsequent analyses. Examination of additionally sampled eleven specimens at Puerto Coquira, Panama Bay, PA (not included in this study), showed that only three individuals had hyperossified frontals and eight were lacking the typical hyperossification
*Cathorops*	*hypophthalmus* (Steindachner 1876)	K^b^ (1)	
	*tuyra* (Meek and Hildebrand 1923)	M^f^ (15)	Species is known to occur in Pacific estuaries and lower reaches of rivers (Fischer et al. 1995). The species has been found in Lake Alajuela and Lake Gatun in the Caribbean watershed, in the latter even being reproductively active (D. Sharpe, pers. comm., December, 2015), but no official report of occurrences of *C. tuyra* on the Atlantic side of Panama has been made. Our sample of *C. tuyra* originates from Puente del Río Chagres, located between the Panama Canal and Lake Alajuela, approx. 59 km inland (distances were calculated following meanders with Google Earth). Therefore, the sampled population can be considered as true freshwater inhabitants
	*fuerthii* (Steindachner 1876)	L^b^ (1), J^b^ (4)	
	sp. indet.	H^b^ (4)	All 4 sequenced individuals had identical *ATPase 8/6* sequence that is not present in the reference dataset, but had sequence affinity to *Cathorops*
	*nuchalis (Günther 1864)	A^f^ (12)	*ATPase 8/6 sequence is identical for both species. Shape data for both species were merged in the phylogenetic analysis as the phylogenetic tree lacks the resolution of both species. They were treated as individual species in PCA (Fig. 3) and CVA (Fig. 5)
	*wayuu (Betancur‐R et al. 2012)	B^b^ (20), C^b^ (14), D^m^ (6), F^m^ (9)	

a Capital letter refers to sampling locality illustrated in Figure 1; small superscript letter refers to habitat characteristic – f: fresh, b: brackish, and m: salt water; and numbers in brackets refer to number of sampled individuals in that specific locality.

### Geometric morphometrics

#### Patterns of opercle shape variation among ariid species

Principal component analysis (PCA) was used to investigate the distribution of opercle shapes of ariid catfishes from different habitats in morphospace. Despite being small (3.8%), the within‐group size variation was removed prior to PCA.

The first three PCs accounted for 75.07% of the observed variation (Fig. 3B). In the morphospace plot, *Bagre* took negative values along PC1 (Fig. 3A) with *B. pinnimaculatus* separating in morphospace (most left cluster) from the other *Bagre* species (Fig. S1), as well as from all other analyzed species. The two *B. pinnimaculatus* populations (inferred from typical hyperossified frontals vs. lack of hyperossified frontals, as well as different *ATPase 8/6* haplotypes) did not overlap in the scatterplot of PC1 versus PC2 and PC1 versus PC3 (data not presented here), with *B. pinnimaculatus* featuring the hyperossification of the frontals forming the lower part of the *B. pinnimaculatus* cohort. PC2 did not distinguish any of the groups in the scatterplot. The least overlap of genera and species in opercle shape was evident in the scatterplot of PC1 versus PC2 (Fig. 3A). *Bagre* and *Cathorops* were separated along PC1, whereas the genera *Ariopsis*,* Sciades*, and *Notarius* overlap in morphospace. The opercle shape changes along PC1 and PC2 followed a dorsal–ventral compression and an anterior–posterior stretching, respectively (Fig. 3A). PC3 demonstrated the shape change from a blunt ventral surface of the posterior edge to a pointy ventral surface.

It became apparent that opercle shapes of the specimens cluster by genus rather than by habitat. To test for phylogenetic signal in our shape data, a phylogeny was plotted on PC scores of species means producing a phylomorphospace. Genera took mainly distinct areas in the phylomorphospace, deviating from the root (Fig. 4A). An apparent phylogenetic structure was tested with the null hypothesis of absence of phylogenetic signal in the morphometric shape data and was rejected (*P* = 0.001). Additionally, Pagel's *λ* of 1 tested on PC1 is not significantly different from 1 (*P* = 1); likewise, *λ* for PC2 is 0.75 (*P* = 0.19), suggesting that opercle shape evolution happened according to the Brownian model and that opercle shape traits are as similar as expected by their phylogenetic distance. The result that opercle shapes were most similar within each genus is visualized in the plot of Procrustes coordinates (shape) on the phylogeny and the ancestral shape reconstruction (Fig. 4B).

#### Opercle shape changes within Ariidae according to habitat

**Figure 5:**
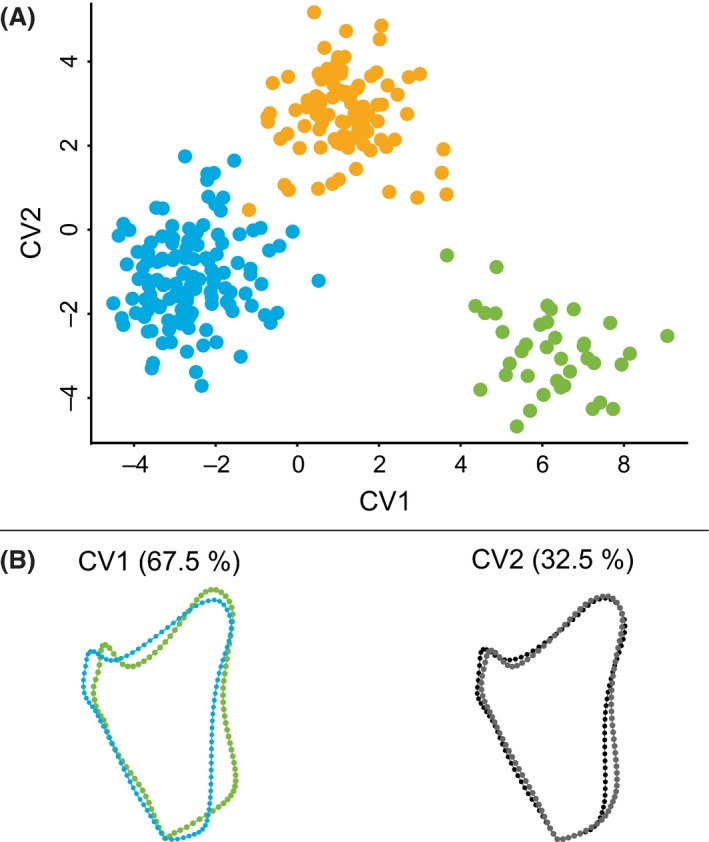
Opercle shape variation in ariid catfish species from marine, brackish, and freshwater habitat. (A) The upper figure shows a scatterplot of individual scores from canonical variate analysis (CVA) comparing ariid catfishes from freshwater (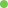), brackish (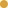), and marine (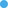) habitat for opercle shape. The first two canonical variates (CVs) capture the entire variance (100%) observed between the three groups. CV1 separates them from negative to positive values, from marine, through brackish, to freshwater species, respectively. (B) Opercle shape changes are presented for CV1 and CV2. For CV1, scale factors −4 (blue, representing marine opercle shape) and +8 (green, representing freshwater opercle shape) were applied, and for CV2, −4 (black) and +4 (gray).

To assess the shape changes that occur among specimens from the sampled habitats (freshwater, brackish, and marine), a CVA was conducted on the specified groups. The first two CVs explained 100% of the observed variation among freshwater, brackish, and marine samples (Fig. 5A). CV1 separated the three habitats, with the marine group exhibiting the most negative values, the brackish group having values smaller than zero, and the freshwater group having positive values. CV2 separated the brackish group from the marine and freshwater group with the first having values larger than zero and the latter having values smaller than zero. All habitats were distinct in shape as measured by both Procrustes and Mahalanobis distances (Table 2). The most pronounced group differences were found in the pairwise comparison of freshwater and marine shape data. The underlying opercle shape change from marine to freshwater habitat (Fig. 5B, CV1) was reflected in a transformation of a rather triangular opercle shape without any distinguishable features to a more complex shape. A ventral protuberance on the posterior edge makes the overall shape of the ventral surface blunter. However, the meeting point of the posterior and anterior edge becomes more pointed. The ventral part of the anterior edge becomes progressively more sigmoidally curved with a deeper notch forming right behind the articular process. The shape change in CV2 was less pronounced although 32.5% of the observed variation is captured along that axis.

**Table 2:** Distance matrices for opercle Procrustes landmark data derived from canonical variate analysis (CVA) of groups defined by habitat occupation

	f	b	m
f	–	7.5831 (<0.0001)	9.0013 (<0.0001)
b	0.0270 (0.0120)	–	5.4069 (<0.0001)
m	0.0642 (<0.0001)	0.0542 (<0.0001)	–

*P*‐values (shown in parenthesis) calculated by 1000 random permutations per test to determine statistical significance of differences between pairs of habitats. f: freshwater; b: brackish; m: marine. Above diagonal is Mahalanobis distances; below diagonal is Procrustes distances.

## Discussion and Conclusion

### Species identification using a mtDNA marker

Molecular species identification led to three main conclusions. First, taxonomic identification using *ATPase 8/6* demonstrates that the taxonomy of tropical American ariid catfishes is still unclear. Based on *ATPase 8/6* haplotypes, *B. pinnimaculatus* seems to contain two distinct taxa. The haplotypes correlate with morphological features of neurocrania that diverged from the description of *B. pinnimaculatus* (Cooke and Jiménez 2008a), one featuring typical hyperossified frontals and one lacking the hyperossified frontals. This is also suggested by subsequent inspection of opercle shape clusters in morphospace. Additional sampling of eleven more individuals from the eastern part of the Gulf of Panama and subsequent maceration of the skulls confirmed our suspicion that the two morphotypes might rather co‐occur than distribute separately along the shore and that the absence of the hyperossification of the frontals is not a rare event. It remains to be validated whether these two morphs represent separate species.

Four individuals from Rio San Pedro, PA, with affinity to *Cathorops* could not be assigned to any known *ATPase 8/6* sequence and remain unidentified. *Notarius biffi*, the distribution of which was believed to be restricted from El Salvador to Costa Rica (Betancur‐R and Acero 2004), was confirmed by molecular analyses to be present south of El Salvador as inferred from distribution records (Robertson and Allen 2015). We therefore strongly recommend the inclusion of molecular‐based identification in similar studies that focus on the taxonomy of ariid catfishes and the description of new species. Wrong assignment of a specimen to a different species biases possible results and conclusions drawn from them.

Second, the DNA sequencing was helpful in showing that the Paraguaná Peninsula, VE, might be a geographic dispersal barrier to populations of marine *S. herzbergii*, as manifested in distinct *ATPase 8/6* haplotypes. Such a geographic barrier was demonstrated for freshwater species from the Venezuelan coast (Rodriguez‐Olarte et al. 2009), but coastal marine species were so far not included in the biogeographic study of species occurrences. Coastal marine species might face similar migration barriers as freshwater species. This is important when considering possible biogeographic scenarios of the initial dispersal of Ariidae in deep time, which is an occurrence throughout the Tethys Sea for this “family” (Betancur‐R et al. 2007). So far the molecular within‐ and between‐species variation has not been assessed in Ariidae, and species from one locality are automatically considered being identical to species in another locality.

Third, we found four recent cases of freshwater invasions where *ATPase 8/6* sequences are still identical between marine/brackish and freshwater populations, as is the case in *C. tuyra*,* N. cookei*, possibly *S. parkeri*, and in the sister species *C. wayuu* and *C. nuchalis*. We note that mitochondrial DNA might fail to detect hybridization or introgression events, and further nuclear‐based evidence is needed to evaluate whether incipient speciation is going on.

*Cathorops. tuyra* has migrated from the Pacific side of Panama, where it inhabits Pacific estuaries and lowland rivers, to the Atlantic watershed of Panama since the termination of the Panama Canal in 1914. It has managed to get through the lock gates of the Panama Canal, which ostensibly were an artificial dispersal barrier, reaching Gatún Lake via the Chagres River, where it now thrives and breeds. It has also been recorded recently in artificial Alajuela Lake, which was formed by damming the River Chagres further upstream in 1934 (D. Sharpe, pers. comm., December 2015). This represents a distance of at least 50 km from the Pacific exit of the Panama Canal. The Chagres River flows into the Atlantic at Fuerte de San Lorenzo, 60 km from our collection station on the Rio Chagres Bridge on the Trans‐Isthmian Highway. On the Pacific watershed, A. Vega (pers. comm., December, 2015) recorded *C. tuyra* as far inland as the Gatún River, an affluent of the Santa Maria River, at the bridge, near San Francisco, approx. 95 km from the Santa Maria marine exit.

*Notarius cookei* was captured in freshwater at Tierra Hueca (76 km inland) and La Raya (69 km) along with *C. tuyra* (A. Vega, pers. comm., December, 2015) (distances were calculated following meanders of the Santa Maria River with Google Earth). In the archaeological record, this species has only been recorded at the boundary of marine influence where surface water has been shown to be fresh (in middens radiocarbon‐dated between 1900 and 1500 radiocarbon years ago at Sitio Sierra, now 13 km from the sea) (Cooke and Jiménez 2008b). A priority for the future is to locate more dated refuse dumps with fish remains further inland in order to clarify *N. cookei's* dispersal pattern through time.

*Cathorops wayuu* (brackish, marine) from the Venezuelan coast and *C. nuchalis* (freshwater) from Maracaibo Lake, VE, are described as being two separate species based on morphology. Their *ATPase 8/6* sequences are identical, though. This species pair might represent an example of freshwater adaptation that is older than in the examples described above, as a disparate morphology has been already noted (Marceniuk et al. 2012).

The identification of *S. parkeri* in the Orinoco River, 320 km inland and outside its known habitat range (Betancur‐R et al. 2008), was unexpected; whether a freshwater population has been established remains to be proven. However, we cannot exclude the possibility that *S. parkeri* was brought from the coast to the fish market although it seems unlikely. No coastal fishes are sold at local markets in the amazon region of Venezuela.

Those four cases are ideal for applying high‐throughput DNA sequencing targeting the marine/brackish and freshwater populations and species to gain insights into the molecular changes associated with adaptations to freshwater environments.

### Geometric morphometrics

#### Patterns of opercle shape variation among ariid species

The present study aimed to identify patterns in ariid opercle shape variation along a major habitat transition from sea to freshwater. Three examples of opercle shape divergences are known so far from the literature: (1) divergence after freshwater invasion in Alaskan *G*. *aculeatus* populations (Kimmel et al. 2008) resulting in the same shape optima in different lakes, (2) decaying phylogenetic signal in major clades with opercle shape divergence along benthic–pelagic axis in subfamilies in Antarctic notothenioids (Wilson et al. 2013a), and (3) adaptation to feeding ecology in Lake Tanganyika cichlids (Wilson et al. 2015).

In this study, we found the same pattern of shape evolution (anterior–posterior stretching and dorsal–ventral compression) of the opercle along major axes of variance as was identified in previous studies. Additionally, we found strong phylogenetic structure in the opercle shape data of the different ariid species, meaning that the closer species were related, the more similar their opercles were, conforming to the Brownian model of evolution. Ariid genera formed separate cohorts in morphospace (Fig. 3). These results underline the importance of taking into consideration phylogenetic relationships in the study of ariid opercle shape. Nonetheless, similar opercle shapes were found when comparing species from one habitat to species from another habitat. This implies the relevance of habitat on opercle shape to a certain degree. We did not detect any subclade divergence as in Antarctic notothenioids.

The three examples from the literature and our examples from Ariidae differ in several aspects, but mainly by clade age, species richness, and the kind of habitat change. This makes each example rather unique in its evolutionary history. *G*. *aculeatus* (stickleback) freshwater populations represent an example of repeated evolution resulting in the same shape optima in the different lakes (Arif et al. 2009; Kimmel et al. 2012a), which formed after the last ice age (Bell and Foster 1994), and are sometimes as young as 1983 in the case of Loberg Lake, USA (Arif et al. 2009). Antarctic notothenioids and Ariidae are both radiations that are millions of years old, 17.1–10 ma (Colombo et al. 2015) and 70–40 ma (Betancur‐R 2010; Betancur‐R et al. 2012), respectively. The radiation of Ariidae was limited by competition (Betancur‐R et al. 2012), which might have constrained shape evolution. Antarctic notothenioids on the other hand, encountered only weak competition (Wilson et al. 2013a), which might have facilitated the decay of phylogenetic signal and allowed for divergence along the benthic–pelagic axis in subfamilies. Finally, Lake Tanganyika cichlids are among the most species‐rich vertebrate adaptive radiations (Salzburger et al. 2014). They seem to have radiated into different trophic niches first and only diversified later along the benthic–limnetic axis (Muschick et al. 2014). This inference is in agreement with the finding that opercle shape correlates with gut length as a proxy for feeding mode rather than habitat (Wilson et al. 2015). Our study of the opercle bone in Ariidae allowed us to identify a different pattern of shape evolution compared to the other studied clades. Additionally, the importance of the opercle bone in Ariidae lies in its taxonomic value and the possibility for comparison with the fossil record as was shown in extinct marine species of the garlike *Saurichthys* (Wilson et al. 2013b).

#### Opercle shape changes within Ariidae according to habitat

Although opercle shapes were more similar among closely related species, opercles from freshwater and marine species are significantly distinct in multivariate analyses. For the creation of the phylomorphospace only species were included that were present in the phylogenetic tree, merging *C. tuyra* brackish and freshwater populations, *S. dowii* brackish and freshwater populations, and *C. nuchalis* (freshwater) and *C. wayuu* (brackish and marine) as one species. Therefore, any possible deviating signal from those contrasting habitat populations is lost in the phylomorphospace plot. However, when treated separately such as in the CVA, the three habitat groups were clearly separated, manifesting in a shape change from a rather triangular opercle shape in marine species to a more complex shape in freshwater species. However, we could not demonstrate whether that signal holds when shape data were corrected for phylogenetic dependence of shape values. In order to truly account for the phylogenetic component in the shape data, in a multivariate analysis such as phylogenetic generalized least squares (pGLS) or phylogenetic ANOVA, a time‐calibrated phylogenetic tree would be needed that contains all analyzed species and populations originating from the different habitats.

To conclude, opercle shape is largely conserved across phylogeny in tropical American Ariidae, with closer‐related species being characterized by more similar opercle shapes. This fact validates the application of opercle shape for taxonomic identification of fossil bones. On the other hand, opercle shape in ariid catfishes appears to reflect adaptations along the marine–freshwater macrohabitat axis. Sticklebacks, Antarctic notothenioids, Lake Tanganyika cichlids, and tropical American Ariidae exhibit the same dorsal–ventral compression and anterior–posterior stretching along major axes of variance, suggesting this to be a general trend in teleosts.

## Conflict of Interest

None declared.

## Supporting information


**Appendix S1**. Extended methods.
**Appendix S2**. Extended results.
**Table S1**. Sampling Locations and definition of habitat of species used in this study.
**Table S2**. Number of individuals per species used in PCA (Fig. 3a) and CVA (Fig. 5), and per habitat in CVA.
**Table S3**. Unique identifiers, sampling locality, and species names for all 263 individuals used in this study.
**Figure S1**. Scatterplots of the first three principal components (PCs) of individual ariid opercle shapes (*N* = 263) from 21 species belonging to the genera *Bagre* (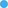), *Sciades* (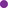), *Cathorops* (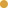), *Notarius* (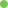) and *Ariopsis* (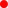).
