# Supplementary material for: Evolution of opercle bone shape along a macrohabitat gradient: species identification using mtDNA and geometric morphometric analyses in neotropical sea catfishes (Ariidae)
Source: Ecol Evol. 2016 Jul 22;6(16):5817–30. doi: 10.1002/ece3.2334 (PMC4983594; doi:10.1002/ece3.2334)
Supplement: Supplementary file 1 — Appendix S1. Extended methods. Appendix S2. Extended results. Table S1. Sampling Locations and definition of habitat of species used in this study. Table S2. Number of individuals per species used in PCA (Fig. 3a) and CVA (Fig. 5), and per habitat in CVA. Table S3. Unique identifiers, sampling locality, and species names for all 263 individuals used in this study. Figure S1. Scatterplots of the first three principal components (PCs) of individual ariid opercle shapes (N = 263) from 21 species belonging to the genera Bagre (), Sciades (), Cathorops (), Notarius () and Ariopsis (). [file ECE3-6-5817-s001.docx]

Supporting Information

Appendix S1. Extended methods.

**Details on *ATPase 8/6* amplification and sequencing conditions.**

For amplification and sequencing of mitochondrial *ATPase 8/6* primers 8.2 L8331 5’-AAAGCRTYRGCCTTTTAAGC-3’ and 3.2 H9236 5’-GTTAGTGGTCAKGGGCTTGGRTC-3’ were used (Betancur-R. *et al.*, 2007). *ATPase 8/6* was amplified using REDTaq® DNA polymerase (Sigma-Aldrich). Cycling conditions were as follows: 1 cycle: 94 °C 2 min; 25 cycles: 94 °C 30 s, 54 °C 40 s and 72 °C 1 min; 1 cycle: 72 °C 10 min. PCR-products were enzymatically purified by using the ExoSAP-IT™ PCR Clean-Up Kit (GE Healthcare). The templates were sequenced on an Applied Biosystems 3130xl Genetic Analyzer (Life Technologies) using the Big Dye® Terminator Ready Reaction Mix 3.1 (Applied Biosystems). Sequencing conditions: 1 cycle: 96 °C 1 min; 25 cycles: 96 °C 15 s, 51°C 15 s, 60 °C 4 min.

Appendix S2. Extended results.

**Species identification using the mtDNA marker *ATPase 8/6*.**

***Sciades***. *S. proops* (Valenciennes, 1840) from the Gulf of Venezuela (marine) and Puerto La Cruz (marine), and *S. herzbergii* (Bloch, 1784) from the Gulf of Venezuela (marine) and Clarines (brackish), VE, clustered according to species, with both species exhibiting two separate clusters of populations according to geographic origin of samples. *S. dowii* (Gill, 1863) specimens from the mouth of Río Hato, Puerto Caimito (brackish) and Río Santa Maria (freshwater), all from the Pacific side of Panama, were identical in *ATPase 8/6* sequence. Two individuals from the Orinoco River (freshwater) bought at a fish market in Ciudad Bolívar, VE, located 320 km inland, were identified as the marine-brackish *S. parkeri* (Traill, 1832). This species is described as occurring in lower parts of rivers from the Gulf of Paria, VE, to Brazil (Betancur-R. *et al.*, 2008).

***Ariopsis***. Brackish *A. seemanni* (Günther, 1864) specimens from Río Estero Salado at the Pacific side of Panama and marine *Ariopsis sp. nov.* (Ricardo Betancur-R., personal communication, May 2014) specimens from Casaya, Pearl Islands, PA, were confirmed.

***Notarius***. *N. cookei* (Acero P. & Betancur-R., 2002) is described as being a brackish species (Betancur-R. *et al.*, 2007). Specimens from Santa Maria River 10 km upstream (in freshwater), PA, were confirmed by the *ATPase 8/6* sequence. *N. kessleri* (Steindachner, 1876), sampled in the estuary of Rio San Pedro, near Montijo, and Rio Estero Salado, PA, were confirmed. A single specimen each of *N. biffi* (Betancur-R. & Acero P., 2004) from the Río San Pedro estuary, and *N. planiceps* from Rio Estero Salado, PA, could be confirmed by *ATPase 8/6* sequence. *N. quadriscutis* (Valenciennes, 1840) from Clarines, VE, and *N. grandicassis* (Valenciennes, 1840) specimens from the Gulf of Venezuela formed a separate clade each. A single individual of

***Bagre***. *B. bagre* (Linnaeus, 1766) from the Gulf of Venezuela (marine) and *B.* aff. *marinus* (marine) from the Gulf of Venezuela and Puerto La Cruz, VE, were confirmed as these species. *B. panamensis* (Gill, 1863) and *B. pinnimaculatus* (Steindachner, 1876) from the Río Estero Salado estuary (brackish), PA (Pacific side) were also confirmed as these species. Samples identified by morphology as *B. pinnimaculatus* from the Gulf of Panama (marine) showed differences in the neurocrania. Several specimens diverged from the morphology of *B.* *pinnimaculatus* by lacking the typical hyperossification of the frontals. They did not exhibit the phenotype of *B. panamensis*, either, the only other *Bagre* species occurring in the Eastern Pacific. Morphologically they resembled *B. bagre* from the Atlantic. In the ML tree those specimens formed a sister clade to *B. pinnimaculatus*. However, due to their molecular relatedness they were considered being *B. pinnimaculatus* in the multivariate analyses.

***Cathorops***. *C. hypophthalmus* (Steindachner, 1876) was verified from the Río Estero Salado estuary (brackish). *C. tuyra* (Meek & Hildebrand, 1923) is known to occur in Pacific estuaries and lower reaches of rivers (Fischer *et al.*, 1995). The species has been found in Lake Alajuela and Lake Gatun, in the latter even being reproductively active (Diana Sharpe, personal communication, December, 2015) but no official report of occurrences of *C. tuyra* on the Atlantic side of Panama has been made. Our sample of *C. tuyra* originates from Puente del Río Chagres, located between the Panama Canal and Lake Alajuela, approx. 59 km inland (distances were calculated following meanders with Google Earth), therefore the sampled population can be considered as true freshwater inhabitants. The *ATPase 8/6* sequences were identical to the reference sequence from specimens collected in brackish water on the Pacific side (in the reference dataset [Betancur-R., 2009]). *C. fuerthii* (Steindachner, 1876) was sampled in the Pacific drainages of Río Parita and Río Hato (brackish), PA, and verified. Four ariid specimens from brackish habitats in the Río San Pedro estuary, PA, were rapidly assigned to *C. steindachneri* (Gilbert & Starks, 1904), *N. cookei*, *C. tuyra* and *C. multiradiatus* (Günther, 1864). However, they all exhibited an identical *ATPase 8/6* sequence that is not present in the reference dataset and are henceforth declared as *C.* sp*.* indet. *C. nuchalis* (Günther, 1864) was caught in the south of Lake Maracaibo (freshwater) near Puerto Concha, Zulia state, VE. *C. wayuu* (Betancur-R., Acero P. & Marceniuk, 2012) was sampled in the drainage of the lake into the Atlantic at Isla de Toa and Isla de San Carlos (brackish) as well as along the Atlantic coast (marine) as far as Puerto la Cruz, VE. The *ATPase 8/6* sequences of both species clustered together. Shape data for both species was merged in the phylogenetic analysis as the phylogenetic tree lacks the resolution of both species. They were treated as individual species in normal multivariate analyses.

Table S1. Sampling Locations and definition of habitat of species used in this study.

| Location | Country | GPS coordinates | Habitat | Species |
| --- | --- | --- | --- | --- |
| Lago de Maracaibo / Isla de Toas (B) | Zulia, Venezuela | 10°57'9.50"N 71°38'49.54"W | brackish | *C. wayuu** |
| Lago de Maracaibo /Isla de San Carlos (B) | Zulia, Venezuela | 10°59'55.1''N 71°36'19.8''W | brackish | *B. bagre* |
| Lago de Maracaibo / Puerto Concha (A) | Zulia, Venezuela | 9°05’46.0”N 71°42´52”W | freshwater | *C. nuchalis** |
| Lago de Maracaibo / Guarico (C) | Zulia, Venezuela | 10°43'52.0''N 71°31'40.2''W | brackish | *C. wayuu** |
| Gulf of Venezuela (D) | Falcon, Venezuela | 11°14'15.3''N 70°30'53.1'' W | marine | *S. proops, S. herzbergii, C. wayuu, B. aff. marinus, B. bagre, N. grandicassis* |
| Clarines (E) | Anzoategui, Venezuela | 10° 3'46.76"N 65°11'5.23"W | brackish | *N. quadriscutis, S. herzbergii, S. proops* |
| Puerto La Cruz (F) | Anzoategui, Venezuela | 10°12'58.63"N 64°38'39.16"W | marine | *C. wayuu*, B. aff. marinus S. proops* |
| Ciudad Bolivar (G) | Bolívar, Venezuela | 8°8'51.46" N 63°32'10.68"W | freshwater | *S. parkeri* |
| Pearl Islands / Casaya island (P) | Panama | 8°34'38.64"N 79°3'3.636" W | marine | *A. nov. sp.* |
| Puente del Rio Chagres (M) | Panama | 9°11'34.66"N 79°39'9.42"W | freshwater | *C. tuyra* |
| Rio Hato (L) | Panama | 8°20'32.4"N 80°09'56.4"W | brackish | *C. fuerthi, S. dowii* |
| Rio Santa Maria (I) | Panama | 8° 6'20.30"N 80°33'16.06"W | freshwater | *N. cookei, S. dowii* |
| Rio Parita (J) | Panama | 8°01'13.69"N 80°27'11.15"W | brackish | *C. fuerthi* |
| Rio Estero Salado (K) | Panama | 8°10'30.324"N 80°29'35.052" W | brackish | *B. pinnimaculatus, B. panamensis, N. planiceps, N. kessleri, C. hypophthalmus, A. seemanni* |
| Rio San Pedro (H) | Panama | 7°50' 59.208"N 81°07' 3.972" W | brackish | *N. kessleri, N. biffi, C.* sp*.* indet. |
| Puerto Caimito (N) | Panama | 8°52'18.88"N 79°42'32.99"W | marine | *S. dowii* |
| Gulf of Panama (O) | Panama | 8°48'56.55"N 79°22'50.85"W | marine | *B. pinnimaculatus* |

Please note that *C. wayuu* und *C. nuchalis* are differentiated here on morphological basis as two separate species although the genetic evidence is missing. Both species together are summarized as *C. sp* in multivariate analyses that depend on a phylogenetic tree. Letters in brackets refer to locations in the sampling map Fig. 1.

Table S2. Number of individuals per species used in PCA (Fig. 3a) and CVA (Fig. 5), and per habitat in CVA.

| Species | Freshwater | Brackish | Marine | total |
| --- | --- | --- | --- | --- |
| *Ans* | 0 | 0 | 9 | 9 |
| *Ase* | 0 | 5 | 0 | 5 |
| *Bba* | 0 | 0 | 5 | 5 |
| *Bma* | 0 | 0 | 19 | 19 |
| *Bpa* | 0 | 3 | 0 | 3 |
| *Bpi* | 0 | 1 | 24 | 25 |
| *Cfu* | 0 | 5 | 0 | 5 |
| *Chy* | 0 | 1 | 0 | 1 |
| *Csp* | 12 | 34 | 15 | 61 |
| *Ctu* | 15 | 0 | 0 | 15 |
| *Cun* | 0 | 4 | 0 | 4 |
| *Nbi* | 0 | 1 | 0 | 1 |
| *Nco* | 7 | 0 | 0 | 7 |
| *Ngr* | 0 | 0 | 11 | 11 |
| *Nke* | 0 | 10 | 0 | 10 |
| *Npl* | 0 | 1 | 0 | 1 |
| *Nqu* | 0 | 10 | 0 | 10 |
| *Sdo* | 2 | 3 | 0 | 5 |
| *She* | 0 | 15 | 31 | 46 |
| *Spa* | 2 | 0 | 0 | 2 |
| *Spr* | 0 | 1 | 17 | 18 |
| total | 38 | 94 | 131 | 263 |

Table S3. Unique identifiers, sampling locality, and species names for all 263 individuals used in this study.

| Identifier | Locality | Species abb. |
| --- | --- | --- |
| 08E2 | Pearl Islands / Casaya island, PA | *A.* sp. nov. |
| 08E3 | Pearl Islands / Casaya island, PA | *A.* sp. nov. |
| 08E4 | Pearl Islands / Casaya island, PA | *A.* sp. nov. |
| 08E5 | Pearl Islands / Casaya island, PA | *A.* sp. nov. |
| 08E6 | Pearl Islands / Casaya island, PA | *A.* sp. nov. |
| 08E8 | Pearl Islands / Casaya island, PA | *A.* sp. nov. |
| 08E9 | Pearl Islands / Casaya island, PA | *A.* sp. nov. |
| 08F1 | Pearl Islands / Casaya island, PA | *A.* sp. nov. |
| 08F2 | Pearl Islands / Casaya island, PA | *A.* sp. nov. |
| 08C6 | Rio Estero Salado, PA | *A. seemanni* |
| 08C7 | Rio Estero Salado, PA | *A. seemanni* |
| 08C8 | Rio Estero Salado, PA | *A. seemanni* |
| 08C9 | Rio Estero Salado, PA | *A. seemanni* |
| 08D1 | Rio Estero Salado, PA | *A. seemanni* |
| 04A8 | Gulf of Venezuela | *B. aff. marinus* |
| 04E9 | Gulf of Venezuela | *B. aff. marinus* |
| 04F1 | Gulf of Venezuela | *B. aff. marinus* |
| 04F2 | Gulf of Venezuela | *B. aff. marinus* |
| 04G5 | Gulf of Venezuela | *B. aff. marinus* |
| 04G6 | Gulf of Venezuela | *B. aff. marinus* |
| 04G7 | Gulf of Venezuela | *B. aff. marinus* |
| 04G8 | Gulf of Venezuela | *B. aff. marinus* |
| 04G9 | Gulf of Venezuela | *B. aff. marinus* |
| 04H6 | Gulf of Venezuela | *B. aff. marinus* |
| 04H7 | Gulf of Venezuela | *B. aff. marinus* |
| 04H8 | Gulf of Venezuela | *B. aff. marinus* |
| 05I5 | Gulf of Venezuela | *B. aff. marinus* |
| 05I6 | Gulf of Venezuela | *B. aff. marinus* |
| 05I7 | Gulf of Venezuela | *B. aff. marinus* |
| 06A2 | Gulf of Venezuela | *B. aff. marinus* |
| 06A6 | Puerto La Cruz, VE | *B. aff. marinus* |
| 06A7 | Puerto La Cruz, VE | *B. aff. marinus* |
| 06A8 | Puerto La Cruz, VE | *B. aff. marinus* |
| 04F5 | Gulf of Venezuela | *B. bagre* |
| 04F6 | Gulf of Venezuela | *B. bagre* |
| 04F7 | Gulf of Venezuela | *B. bagre* |
| 05A8 | Lago de Maracaibo /Isla de San Carlos, VE | *B. bagre* |
| 05A9 | Lago de Maracaibo /Isla de San Carlos, VE | *B. bagre* |
| 08B8 | Rio Estero Salado, PA | *B. panamensis* |
| 08B9 | Rio Estero Salado, PA | *B. panamensis* |
| 08C1 | Rio Estero Salado, PA | *B. panamensis* |
| 01A1 | Gulf of Panama | *B. pinnimaculatus** |
| 01A3 | Gulf of Panama | *B. pinnimaculatus** |
| 01A5 | Gulf of Panama | *B. pinnimaculatus** |
| 01A7 | Gulf of Panama | *B. pinnimaculatus** |
| 01A9 | Gulf of Panama | *B. pinnimaculatus** |
| 01B2 | Gulf of Panama | *B. pinnimaculatus** |
| 01B4 | Gulf of Panama | *B. pinnimaculatus** |
| 01B6 | Gulf of Panama | *B. pinnimaculatus* |
| 01B8 | Gulf of Panama | *B. pinnimaculatus** |
| 01C1 | Gulf of Panama | *B. pinnimaculatus** |
| 01C3 | Gulf of Panama | *B. pinnimaculatus** |
| 01C5 | Gulf of Panama | *B. pinnimaculatus* |
| 01C8 | Gulf of Panama | *B. pinnimaculatus* |
| 01C9 | Gulf of Panama | *B. pinnimaculatus* |
| 01D2 | Gulf of Panama | *B. pinnimaculatus** |
| 01D4 | Gulf of Panama | *B. pinnimaculatus** |
| 01D7 | Gulf of Panama | *B. pinnimaculatus** |
| 01D8 | Gulf of Panama | *B. pinnimaculatus** |
| 01E1 | Gulf of Panama | *B. pinnimaculatus** |
| 01E3 | Gulf of Panama | *B. pinnimaculatus** |
| 01E5 | Gulf of Panama | *B. pinnimaculatus** |
| 01E8 | Gulf of Panama | *B. pinnimaculatus** |
| 01E9 | Gulf of Panama | *B. pinnimaculatus** |
| 01F2 | Gulf of Panama | *B. pinnimaculatus** |
| 08B7 | Rio Estero Salado, PA | *B. pinnimaculatus* |
| 08D3 | Rio Parita | *C. fuerthi* |
| 08D4 | Rio Parita | *C. fuerthi* |
| 08D5 | Rio Parita | *C. fuerthi* |
| 08D6 | Rio Parita | *C. fuerthi* |
| 08D7 | Rio Parita | *C. fuerthi* |
| 08C4 | Rio Estero Salado, PA | *C. hypophthalmus* |
| 01F4 | Lago de Maracaibo / Puerto Concha, VE | *C. nuchalis* |
| 01F5 | Lago de Maracaibo / Puerto Concha, VE | *C. nuchalis* |
| 01F6 | Lago de Maracaibo / Puerto Concha, VE | *C. nuchalis* |
| 01F7 | Lago de Maracaibo / Puerto Concha, VE | *C. nuchalis* |
| 01G1 | Lago de Maracaibo / Puerto Concha, VE | *C. nuchalis* |
| 01G2 | Lago de Maracaibo / Puerto Concha, VE | *C. nuchalis* |
| 01G8 | Lago de Maracaibo / Puerto Concha, VE | *C. nuchalis* |
| 01H6 | Lago de Maracaibo / Puerto Concha, VE | *C. nuchalis* |
| 01H9 | Lago de Maracaibo / Puerto Concha, VE | *C. nuchalis* |
| 07A1 | Lago de Maracaibo / Puerto Concha, VE | *C. nuchalis* |
| 07A6 | Lago de Maracaibo / Puerto Concha, VE | *C. nuchalis* |
| 07B2 | Lago de Maracaibo / Puerto Concha, VE | *C. nuchalis* |
| 08A4 | Rio San Pedro, PA | *C.* sp. indet. |
| 08A5 | Rio San Pedro, PA | *C.* sp. indet. |
| 08A6 | Rio San Pedro, PA | *C.* sp. indet. |
| 08B5 | Rio San Pedro, PA | *C.* sp. indet. |
| 08F3 | Puente del Rio Chagres, PA | *C. tuyra* |
| 08F4 | Puente del Rio Chagres, PA | *C. tuyra* |
| 08F5 | Puente del Rio Chagres, PA | *C. tuyra* |
| 08F6 | Puente del Rio Chagres, PA | *C. tuyra* |
| 08F7 | Puente del Rio Chagres, PA | *C. tuyra* |
| 08F8 | Puente del Rio Chagres, PA | *C. tuyra* |
| 08F9 | Puente del Rio Chagres, PA | *C. tuyra* |
| 08G1 | Puente del Rio Chagres, PA | *C. tuyra* |
| 08G2 | Puente del Rio Chagres, PA | *C. tuyra* |
| 08G3 | Puente del Rio Chagres, PA | *C. tuyra* |
| 08G4 | Puente del Rio Chagres, PA | *C. tuyra* |
| 08G5 | Puente del Rio Chagres, PA | *C. tuyra* |
| 08G6 | Puente del Rio Chagres, PA | *C. tuyra* |
| 08G7 | Puente del Rio Chagres, PA | *C. tuyra* |
| 08G8 | Puente del Rio Chagres, PA | *C. tuyra* |
| 03G1 | Puerto La Cruz, VE | *C. wayuu* |
| 03G3 | Puerto La Cruz, VE | *C. wayuu* |
| 03G5 | Puerto La Cruz, VE | *C. wayuu* |
| 03G6 | Puerto La Cruz, VE | *C. wayuu* |
| 03G7 | Puerto La Cruz, VE | *C. wayuu* |
| 03G8 | Puerto La Cruz, VE | *C. wayuu* |
| 03G9 | Puerto La Cruz, VE | *C. wayuu* |
| 03H1 | Puerto La Cruz, VE | *C. wayuu* |
| 03H2 | Puerto La Cruz, VE | *C. wayuu* |
| 04A5 | Gulf of Venezuela | *C. wayuu* |
| 04A6 | Gulf of Venezuela | *C. wayuu* |
| 04A9 | Gulf of Venezuela | *C. wayuu* |
| 04B1 | Gulf of Venezuela | *C. wayuu* |
| 04F3 | Gulf of Venezuela | *C. wayuu* |
| 04F4 | Gulf of Venezuela | *C. wayuu* |
| 05B1 | Lago de Maracaibo / Guarico | *C. wayuu* |
| 05B3 | Lago de Maracaibo / Guarico | *C. wayuu* |
| 05B4 | Lago de Maracaibo / Guarico | *C. wayuu* |
| 05B6 | Lago de Maracaibo / Guarico | *C. wayuu* |
| 05B7 | Lago de Maracaibo / Guarico | *C. wayuu* |
| 05B8 | Lago de Maracaibo / Guarico | *C. wayuu* |
| 05B9 | Lago de Maracaibo / Guarico | *C. wayuu* |
| 05C1 | Lago de Maracaibo / Guarico | *C. wayuu* |
| 05C2 | Lago de Maracaibo / Guarico | *C. wayuu* |
| 05C6 | Lago de Maracaibo / Guarico | *C. wayuu* |
| 05C7 | Lago de Maracaibo / Guarico | *C. wayuu* |
| 05C8 | Lago de Maracaibo / Guarico | *C. wayuu* |
| 05D2 | Lago de Maracaibo / Guarico | *C. wayuu* |
| 05D3 | Lago de Maracaibo / Guarico | *C. wayuu* |
| 05F3 | Lago de Maracaibo / Isla de Toas | *C. wayuu* |
| 05F4 | Lago de Maracaibo / Isla de Toas | *C. wayuu* |
| 05F5 | Lago de Maracaibo / Isla de Toas | *C. wayuu* |
| 05F6 | Lago de Maracaibo / Isla de Toas | *C. wayuu* |
| 05F7 | Lago de Maracaibo / Isla de Toas | *C. wayuu* |
| 05F8 | Lago de Maracaibo / Isla de Toas | *C. wayuu* |
| 05F9 | Lago de Maracaibo / Isla de Toas | *C. wayuu* |
| 05G1 | Lago de Maracaibo / Isla de Toas | *C. wayuu* |
| 05G2 | Lago de Maracaibo / Isla de Toas | *C. wayuu* |
| 05G4 | Lago de Maracaibo / Isla de Toas | *C. wayuu* |
| 05G5 | Lago de Maracaibo / Isla de Toas | *C. wayuu* |
| 05G6 | Lago de Maracaibo / Isla de Toas | *C. wayuu* |
| 05G7 | Lago de Maracaibo / Isla de Toas | *C. wayuu* |
| 05G8 | Lago de Maracaibo / Isla de Toas | *C. wayuu* |
| 05G9 | Lago de Maracaibo / Isla de Toas | *C. wayuu* |
| 05H1 | Lago de Maracaibo / Isla de Toas | *C. wayuu* |
| 05H2 | Lago de Maracaibo / Isla de Toas | *C. wayuu* |
| 05H3 | Lago de Maracaibo / Isla de Toas | *C. wayuu* |
| 05H4 | Lago de Maracaibo / Isla de Toas | *C. wayuu* |
| 05H5 | Lago de Maracaibo / Isla de Toas | *C. wayuu* |
| 08A9 | Rio San Pedro, PA | *N. biffi* |
| 08G9 | Rio Santa Maria, PA | *N. cookei* |
| 08H3 | Rio Santa Maria, PA | *N. cookei* |
| 08H4 | Rio Santa Maria, PA | *N. cookei* |
| 08H5 | Rio Santa Maria, PA | *N. cookei* |
| 08H6 | Rio Santa Maria, PA | *N. cookei* |
| 08H7 | Rio Santa Maria, PA | *N. cookei* |
| 08H8 | Rio Santa Maria, PA | *N. cookei* |
| 04F8 | Gulf of Venezuela | *N. grandicassis* |
| 04F9 | Gulf of Venezuela | *N. grandicassis* |
| 04G1 | Gulf of Venezuela | *N. grandicassis* |
| 04G2 | Gulf of Venezuela | *N. grandicassis* |
| 04G4 | Gulf of Venezuela | *N. grandicassis* |
| 04H1 | Gulf of Venezuela | *N. grandicassis* |
| 04H4 | Gulf of Venezuela | *N. grandicassis* |
| 04H5 | Gulf of Venezuela | *N. grandicassis* |
| 05H9 | Gulf of Venezuela | *N. grandicassis* |
| 05I1 | Gulf of Venezuela | *N. grandicassis* |
| 05I2 | Gulf of Venezuela | *N. grandicassis* |
| 08A1 | Rio San Pedro, PA | *N. kessleri* |
| 08A2 | Rio San Pedro, PA | *N. kessleri* |
| 08A3 | Rio San Pedro, PA | *N. kessleri* |
| 08A7 | Rio San Pedro, PA | *N. kessleri* |
| 08A8 | Rio San Pedro, PA | *N. kessleri* |
| 08B1 | Rio San Pedro, PA | *N. kessleri* |
| 08B3 | Rio San Pedro, PA | *N. kessleri* |
| 08B4 | Rio San Pedro, PA | *N. kessleri* |
| 08C3 | Rio Estero Salado, PA | *N. kessleri* |
| 08C5 | Rio Estero Salado, PA | *N. kessleri* |
| 08C2 | Rio Estero Salado, PA | *N. planiceps* |
| 03E7 | Clarines, VE | *N. quadriscutis* |
| 03E8 | Clarines, VE | *N. quadriscutis* |
| 03E9 | Clarines, VE | *N. quadriscutis* |
| 03F1 | Clarines, VE | *N. quadriscutis* |
| 03F3 | Clarines, VE | *N. quadriscutis* |
| 03F4 | Clarines, VE | *N. quadriscutis* |
| 03F5 | Clarines, VE | *N. quadriscutis* |
| 03F7 | Clarines, VE | *N. quadriscutis* |
| 03F8 | Clarines, VE | *N. quadriscutis* |
| 03F9 | Clarines, VE | *N. quadriscutis* |
| 08D8 | Puerto Caimito, PA | *S. dowii* |
| 08D9 | Puerto Caimito, PA | *S. dowii* |
| 08E1 | Rio Hato, PA | *S. dowii* |
| 08H1 | Rio Santa Maria, PA | *S. dowii* |
| 08H2 | Rio Santa Maria, PA | *S. dowii* |
| 03A8 | Clarines, VE | *S. herzbergii* |
| 03C1 | Clarines, VE | *S. herzbergii* |
| 03C4 | Clarines, VE | *S. herzbergii* |
| 03C5 | Clarines, VE | *S. herzbergii* |
| 03C6 | Clarines, VE | *S. herzbergii* |
| 03C7 | Clarines, VE | *S. herzbergii* |
| 03C8 | Clarines, VE | *S. herzbergii* |
| 03C9 | Clarines, VE | *S. herzbergii* |
| 03D1 | Clarines, VE | *S. herzbergii* |
| 03D2 | Clarines, VE | *S. herzbergii* |
| 03D3 | Clarines, VE | *S. herzbergii* |
| 03D4 | Clarines, VE | *S. herzbergii* |
| 03D5 | Clarines, VE | *S. herzbergii* |
| 03D6 | Clarines, VE | *S. herzbergii* |
| 03D7 | Clarines, VE | *S. herzbergii* |
| 04A7 | Gulf of Venezuela | *S. herzbergii* |
| 04B3 | Gulf of Venezuela | *S. herzbergii* |
| 04B4 | Gulf of Venezuela | *S. herzbergii* |
| 04B5 | Gulf of Venezuela | *S. herzbergii* |
| 04B6 | Gulf of Venezuela | *S. herzbergii* |
| 04B7 | Gulf of Venezuela | *S. herzbergii* |
| 04B8 | Gulf of Venezuela | *S. herzbergii* |
| 04B9 | Gulf of Venezuela | *S. herzbergii* |
| 04C3 | Gulf of Venezuela | *S. herzbergii* |
| 04C4 | Gulf of Venezuela | *S. herzbergii* |
| 04C5 | Gulf of Venezuela | *S. herzbergii* |
| 04C7 | Gulf of Venezuela | *S. herzbergii* |
| 04C8 | Gulf of Venezuela | *S. herzbergii* |
| 04C9 | Gulf of Venezuela | *S. herzbergii* |
| 04D1 | Gulf of Venezuela | *S. herzbergii* |
| 04D3 | Gulf of Venezuela | *S. herzbergii* |
| 04D4 | Gulf of Venezuela | *S. herzbergii* |
| 04D5 | Gulf of Venezuela | *S. herzbergii* |
| 04D6 | Gulf of Venezuela | *S. herzbergii* |
| 04D7 | Gulf of Venezuela | *S. herzbergii* |
| 04D8 | Gulf of Venezuela | *S. herzbergii* |
| 04D9 | Gulf of Venezuela | *S. herzbergii* |
| 04E1 | Gulf of Venezuela | *S. herzbergii* |
| 04E2 | Gulf of Venezuela | *S. herzbergii* |
| 04E3 | Gulf of Venezuela | *S. herzbergii* |
| 04E4 | Gulf of Venezuela | *S. herzbergii* |
| 04E5 | Gulf of Venezuela | *S. herzbergii* |
| 04E6 | Gulf of Venezuela | *S. herzbergii* |
| 04E7 | Gulf of Venezuela | *S. herzbergii* |
| 04E8 | Gulf of Venezuela | *S. herzbergii* |
| 04H3 | Gulf of Venezuela | *S. herzbergii* |
| 06B9 | Ciudad Bolivar, VE | *S. parkeri* |
| 06C1 | Ciudad Bolivar, VE | *S. parkeri* |
| 03A2 | Clarines, VE | *S. proops* |
| 04A1 | Gulf of Venezuela | *S. proops* |
| 04A2 | Gulf of Venezuela | *S. proops* |
| 04A3 | Gulf of Venezuela | *S. proops* |
| 04A4 | Gulf of Venezuela | *S. proops* |
| 04G3 | Gulf of Venezuela | *S. proops* |
| 04H2 | Gulf of Venezuela | *S. proops* |
| 04H9 | Gulf of Venezuela | *S. proops* |
| 05H6 | Gulf of Venezuela | *S. proops* |
| 05H7 | Gulf of Venezuela | *S. proops* |
| 05H8 | Gulf of Venezuela | *S. proops* |
| 05I3 | Gulf of Venezuela | *S. proops* |
| 05I8 | Gulf of Venezuela | *S. proops* |
| 05I9 | Gulf of Venezuela | *S. proops* |
| 06A1 | Gulf of Venezuela | *S. proops* |
| 06A3 | Gulf of Venezuela | *S. proops* |
| 06A4 | Gulf of Venezuela | *S. proops* |
| 06B1 | Puerto La Cruz, VE | *S. proops* |

* *ATPase 8/6* sequence, and neurocranium diverges from *B. pinnimaculatus*


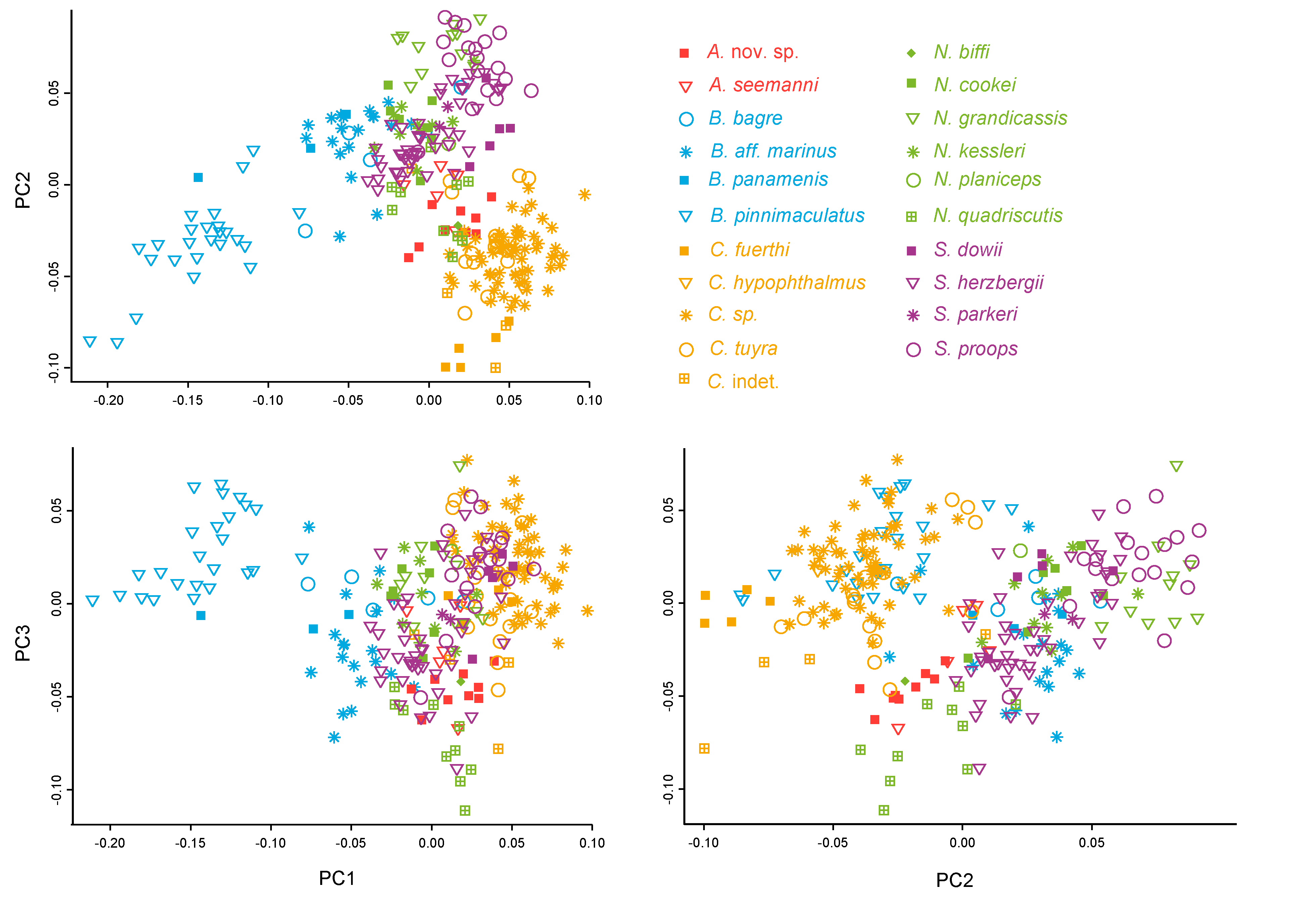


Figure S1. Scatterplots of the first three principal components (PCs) of individual ariid opercle shapes (N=263) from 21 species belonging to the genera *Bagre* (•), *Sciades* (•), *Cathorops* (•), *Notarius* (•) and *Ariopsis* (•). Please note that *C. wayuu* und *C. nuchalis* are pooled here as *C. sp*.

**References**

Betancur-R., R., & Acero, A. 2004. Description of *Notarius biffi* n. sp. and redescription of *N. insculptus* (Jordan and Gilbert) (Siluriformes: Ariidae) from the eastern Pacific, with evidence of monophyly and limits of *Notarius*. *Zootaxa*, **703**: 1–20.

Betancur-R., R., Acero P., A., Bermingham, E. & Cooke, R. 2007. Systematics and biogeography of New World sea catfishes (Siluriformes: Ariidae) as inferred from mitochondrial, nuclear, and morphological evidence. *Mol. Phylogenet. Evol.* **45**: 339–357.

Betancur-R., R., Acero P., A., Bermingham, E. & Cooke, R. 2007. Systematics and biogeography of New World sea catfishes (Siluriformes: Ariidae) as inferred from mitochondrial, nuclear, and morphological evidence. *Mol. Phylogenet. Evol.* **45**: 339–357.

Betancur-R., R., Marceniuk, A.P. & Béarez, P. 2008. Taxonomic Status and Redescription of the Gillbacker Sea Catfish (Siluriformes: Ariidae: *Sciades parkeri*). *Copeia* **4**: 827–834.

Fischer, W., Krupp, F., Schneider, W., Sommer, C., Carpenter, K.E. & Niem, V.H. 1995. *Guia FAO para la identificacion de especies para los fines de la pesca, pacifico centro-oriental Volumen II. Vertebrados - Parte 1*. FAO, Roma.
